# Application of Visible and Near-Infrared Hyperspectral Imaging to Determine Soluble Protein Content in Oilseed Rape Leaves

**DOI:** 10.3390/s150716576

**Published:** 2015-07-09

**Authors:** Chu Zhang, Fei Liu, Wenwen Kong, Yong He

**Affiliations:** College of Biosystems Engineering and Food Science, Zhejiang University, 866 Yuhangtang Road, Hangzhou 310058, China; E-Mails: chuzh@zju.edu.cn (C.Z.); zjukww@163.com (W.K.)

**Keywords:** hyperspectral imaging, soluble protein content, weighted regression coefficient, successive projections algorithm, genetic algorithm-partial least squares

## Abstract

Visible and near-infrared hyperspectral imaging covering spectral range of 380–1030 nm as a rapid and non-destructive method was applied to estimate the soluble protein content of oilseed rape leaves. Average spectrum (500–900 nm) of the region of interest (ROI) of each sample was extracted, and four samples out of 128 samples were defined as outliers by Monte Carlo-partial least squares (MCPLS). Partial least squares (PLS) model using full spectra obtained dependable performance with the correlation coefficient (*r_p_*) of 0.9441, root mean square error of prediction (RMSEP) of 0.1658 mg/g and residual prediction deviation (RPD) of 2.98. The weighted regression coefficient (*Bw*), successive projections algorithm (SPA) and genetic algorithm-partial least squares (GAPLS) selected 18, 15, and 16 sensitive wavelengths, respectively. SPA-PLS model obtained the best performance with *r_p_* of 0.9554, RMSEP of 0.1538 mg/g and RPD of 3.25. Distribution of protein content within the rape leaves were visualized and mapped on the basis of the SPA-PLS model. The overall results indicated that hyperspectral imaging could be used to determine and visualize the soluble protein content of rape leaves.

## 1. Introduction

Seed variety, growth environment, and field management are major concerns in crop growth. The acquisition of spatial and temporal variability of crop growth is one of the goals in precision agriculture (PA) [1]. The nutrient condition detection and the disease diagnosis have significant meaning for crop growth. The growth status of different growth stages could influence the yield of the crops, and the physiological indexes like leaf chlorophyll content, soluble sugar content, soluble protein content, enzyme activity, and others affecting crop growth could be used to understand the crop growth status.

Soluble protein is a valuable indicator of plant physiology, microorganisms, and food [2,3,4,5,6,7,8]. Soluble protein in plants is mainly related to the quality of the edible part and the capacity of resistance of stresses as drought [2], freezing [3], disease [4], salt [5], heavy metal [6], *etc.* Laboratory-based chemical analyses are the most common methods for soluble protein content measurement in plants (Folin-phenol reagent method [9], coomassie brilliant blue method [10], Lowry method [11] and high-performance liquid chromatography (HPLC) method [12]), these kinds of methods are time-consuming, chemical reagent consuming, inefficient and complicated to operate. An accurate determination of soluble protein content on the large scale of fields needs a lot of work for chemical analysis. A rapid, nondestructive method that fits the demand of field application should be developed.

Spectroscopy technique has been applied to estimate the soluble protein content as a rapid and nondestructive method [13,14,15]. Developed from remote sensing, hyperspectral imaging is a novel technique which integrates both spectroscopy and imaging techniques. Hyperspectral imaging acquires both spectral and spatial information simultaneously, which provides a three–dimensional (3D) hypercube of data with a two-dimensional image (spatial) and the spectral data as the third dimension. Gray-scale images are captured at each waveband, and a hyperspectral image is formed by numerous gray-scale images at contiguous discrete spectral bands. As a result, each pixel within the image gets full range spectrum. The spectral and spatial information could be used for quantitative prediction of chemical compositions and physical parameters. With the advantage of acquiring spectral and spatial information of each pixel within the image, hyperspectral imaging provides the capability to map the distribution of quality parameters of samples. The objective of this study is to explore the feasibility of determining soluble protein content of rape leaves by using hyperspectral imaging and visualizing soluble protein distribution of oilseed rape leaves. The specific objectives are to: (1) build robust and accurate calibration models for soluble protein content estimation; (2) determine the superior variable selection method and the corresponding sensitive wavelengths; (3) visualize the soluble protein content in oilseed rape leaf by the optimized calibration model.

## 2. Materials and Methods

### 2.1. Sample Preparation 

The studied cultivar was *Brassica napus* L., cv. ZS758. Oilseed rape were planted in the experimental farm of Zijingang campus, Zhejiang University, Hangzhou, China (30.30° N, 120.08° E). The rapeseeds were sowed in the seedbed on a sunny day in early October, 2012, and the rape seedlings were transplanted to the experimental field in 11 November 2012. In total, 32 plots were prepared for the transplanted seedlings, including two reference plots without any fertilizer and 30 plots with different amounts of nitrogen, phosphorus and potassium acquired by a quadratic orthogonal regression design [16]. Each plot was designed as 1.5 m long and 1.2 m wide including 20 transplanted plants. Four similar experiments were conducted during four growth stage of oilseed rapes: the seedling stage (8 December 2012), the bolting stage (5 March 2013), the florescence stage (20 March 2013), and the pod stage (11 April 2013). For each growth stage, five leaves collected from five plants of each plot at the same leaf position were regarded as one sample, and totally 32 samples with 160 leaves of 32 plots were acquired. In total, 128 samples were collected. The collected rape leaves were taken to the laboratory in an incubator filled with ice and cleaned for image acquisition and the following chemical analysis.

### 2.2. Chemical Analysis of Soluble Protein Content

Coomassie brilliant blue method was applied to measure the reference soluble protein content of rape leaves. A 0.1 g sample was cut from each rape leaf sample avoiding the leaf veins, and ground in a mortar with 1 mL Phosphate Buffer solution (PBS, pH = 7.8), then the grinding fluid was transferred to a 10 mL centrifuge tube. The mortar was washed twice with 2 mL PBS to minimize residual of grinding fluid, the washing fluid was also transferred to the 10 mL centrifuge tube. Then the centrifuge tube was centrifuged at the speed of 10,000 rpm for 15 min at 4 °C. After centrifugation, the supernatant was transferred to a new centrifuge tube, and the new tubes were place in the icebox. 0.1 mL supernatant in the new tube was transferred to a new centrifuge tube, and 5 mL Coomassie brilliant blue G-250 was then added into the new tube and mixed thoroughly. Two minutes later, the thoroughly mixed solution was put into a cuvette for colorimetric analysis at 595 nm by an UV 2450 spectrophotometer (Shimadzu Scientific Instruments, Carlsbad, CA, USA) [10]. The soluble protein content in the tube was calculated by using the linear equation obtained by the standards. The equation was *y* = 147.97*x* − 17.139, where *x* was the absorbance at 595 nm. Then the soluble protein contents of the rape leaves were acquired. For each sample, the average value of three chemical measurements was used as the reference for soluble protein content.

### 2.3. Hyperspectral Imaging System and Image Acquisition

**Figure 1:**
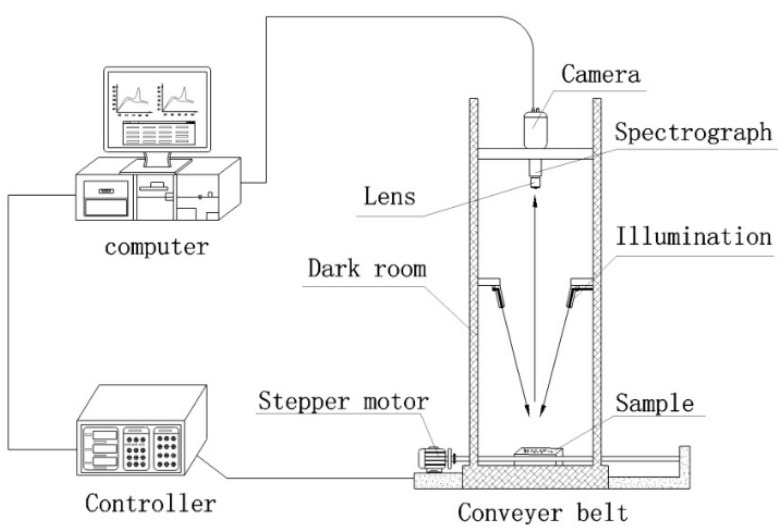
The schematic diagram of the hyperspectral imaging system.

A visible and near-infrared hyperspectral imaging system including the following devices was used: an imaging spectrograph (ImSpector V10E; Spectral Imaging Ltd., Oulu, Finland) covering the spectral range of 380–1030 nm with the spectral resolution of 2.8 nm, a high performance CCD camera (Hamamatsu, Hamamatsu City, Japan) with 672 × 512 (spatial × spectral) pixels, a camera lens (OLES23; Specim, Spectral Imaging Ltd., Oulu, Finland), two 150 W tungsten halogen lamps (Fiber-Lite DC950 Illuminator; Dolan Jenner Industries Inc., Boxborough, MA, USA) for illumination, a conveyer belt driven by a stepper motor (Isuzu Optics Corp, Taiwan, China). All these devices were integrated as the hyperspectral imaging system (Figure 1) and the hyperspectral imaging system was controlled by a data acquisition and system control software (Spectral Image-V10E, Isuzu Optics Corp, Taiwan, China). The samples were scanned line by line along the Y-axis with the sample moving along the X-axis at a certain speed to obtain a three-dimensional hypercube.

To acquire clear and nondeformable hyperspectral images, the moving speed of the conveyer belt, the exposure time of the camera, and the height between the lens of the camera and the sample were adjusted to 2.05 mm/s, 0.13 s, and 36 cm, respectively. 

The original raw images could not be analyzed directly and should be corrected to reflectance hyperspectral images for further processing. The raw image was corrected with the white reference image and the dark image which captured the hyperspectral imaging system under the same condition of acquiring raw images, where the dark reference image was captured by turning off the light source and covering the camera lens thoroughly with its opaque cap, and the white reference image was acquired using a white Teflon tile. The calibrated image *Ic* was calculated using the following equation:

(1) $$I_{c}=\frac{I_{raw}-B}{W-B}$$

where *I_raw_* was the raw hyperspectral image, *B* was the dark reference image, *W* was the white reference image, and *Ic* was the calibrated hyperspectral image.

To extract the spectral information of the sample from the hyperspectral image, the sample region was segmented from the background. Masks were built by ENVI 4.6 (ITT, Visual Information Solutions, Boulder, CO, USA) and Matlab R2009b (The Math Works, Natick, MA, USA). After mask, the reflectance value of the background was set as 0, and the sample region was extracted from the background. 

### 2.4. Spectral Data Extraction

After the image segmentation, the sample region was segmented from the background, and then the ROI should be defined. There are two main methods to define the ROI, one is taking the entire sample region as ROI, and the other is picking part of the sample region. In this study, the sample region of all five leaves was used as ROI. The reflectance values of all pixels in the ROI were averaged as the reflectance spectrum of the sample.

### 2.5. Image Visualization and Distribution Map

In practical application, the quality parameters measured from a small part of the sample were used to represent the quality of the whole sample. However, this has dramatic drawbacks unless we know the distribution of quality parameters over the sample. It was impossible to obtain the precise quality parameters of every pixel within a sample by chemical analysis methods, but the quality parameters of every pixel could be predicted by a precise calibration model, and a prediction map could be drawn by using the prediction value of all pixels in the ROI. A simple, robust, and accurate calibration model could be used to visualize and map the chemical constitution distribution.

### 2.6. Multivariate Data Analysis

Multivariate data analysis was applied to predict soluble protein content in rape leaves using the extracted spectral data from the hyperspectral images. Monte Carlo Partial least square (MCPLS) was used to detect outlier samples [17]. One thousand PLS models were built by randomly selecting 80% of the samples as the calibration set and the remaining 20% samples as the prediction set. The number of PLS models should be large enough to ensure that every sample was selected into the prediction set for certain times. A set of predictive residual errors (PRE) of each sample were obtained. Samples with high mean value of PRE (MPRE) and high standard deviation of PRE (STDPRE) were manually defined as outliers and removed from the samples.

Partial least square regression was a widely used regression method [18,19]. It compressed the spectral data into a few orthogonal variables (called latent variables, LVs) which carry the most information and maximize covariance between the spectral data and the chemical constitutions. In this study, PLS was used to build calibration models with full cross validation to predict the soluble protein content in rape leaves.

### 2.7. Sensitive Wavelengths Selection 

Hyperspectral images acquired in this study were formed by 512 gray-scale images at 512 wavelength bands in the spectral range of 380–1030 nm. The spectral and spatial information of all 512 bands contained redundancy and collinearity, and the large amount of data were difficult to deal with. Sensitive wavelengths were selected from the original or preprocessed wavelengths and could be directly used for developing on-line or portable multispectral equipment. The selected sensitive wavelengths could reduce the computation complexity, improve the predictive ability of calibration models, and simplify the calibration models [20]. The calibration models developed by sensitive wavelengths provided the feasibility to visualize the chemical constitutions within the sample in a fast and simple way. In this study, weighted regression coefficients (*Bw*), genetic algorithm–partial least square (GAPLS) and successive projections algorithm (SPA) were used for sensitive wavelength selection.

#### 2.7.1. Weighted Regression Coefficients

Weighted regression coefficients (*Bw*) were obtained from PLS model [18,21]. Weighted regression coefficient of each wavelength in the PLS model indicated the importance of the wavelength in the model. Wavelengths with large absolute value of weighted regression coefficients were considered to be more relevant for effective prediction, and could be selected as sensitive wavelengths.

#### 2.7.2. Genetic Algorithm-Partial Least Squares

Genetic algorithm-partial least squares (GAPLS) was an effective variable selection method proposed by Leardi [22,23]. The basic principle of genetic algorithm-partial least square used for wavelength selection was that the genetic algorithm was used to select candidate sensitive wavelengths and PLS was applied to evaluate the selected wavelengths. In this study, 100 short runs of GAPLS were applied, and the sensitive wavelengths selected in each run were recorded, and the weighted frequency of each wavelength selected in 100 runs was calculated. The most frequently selected wavelengths were defined as sensitive wavelengths.

#### 2.7.3. Successive Projections Algorithm

Successive projections algorithm (SPA) was a feed forward variable selection method [24,25]. SPA selected the wavelength variables with minimum redundancy and collinearity by projecting one variable on the other variables, and the variable with maximum projection vector was selected as the candidate sensitive wavelength. The sensitive wavelengths were finally selected according to the best multiple linear regression (MLR) model using different candidate sensitive wavelengths.

### 2.8. Model Evaluation

The performances of the calibration models were evaluated in terms of the correlation coefficient (*r*) of cross validation (*r_cv_*), calibration (*r_c_*) and prediction (*r_p_*), root mean square error (RMSE) of cross validation (RMSECV), calibration (RMSEC) and prediction (RMSEP), and the residual prediction deviation (RPD). The model with high *r_cv_*, *r_c_*, *r_p_* and RPD, low RMSECV, RMSEC, and RMSEP was preferred. Particularly, the RPD values between 1.8 and 2.0 indicated a good model, between 2.0–2.5 indicated a very good model and over 2.5 meant that the model performed excellent [18,26].

## 3. Results and Discussion

### 3.1. Spectral Feature of Rape Leaves

**Figure 2:**
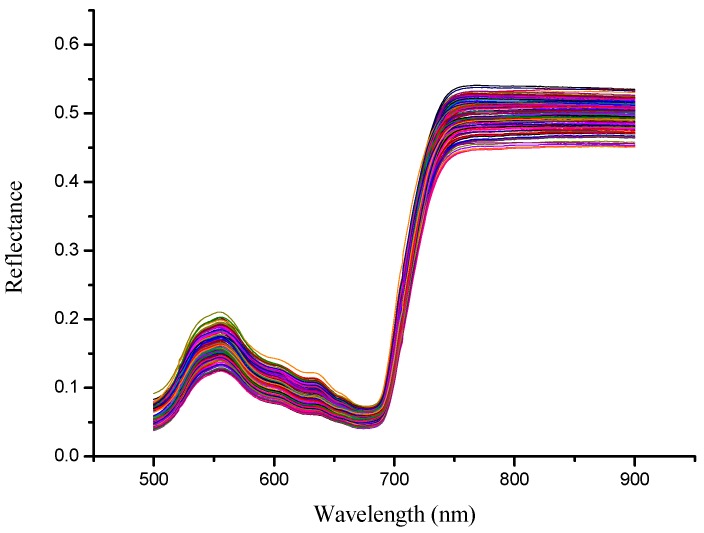
Raw spectra (500–900 nm) of rape leaves samples.

The spectral range 500–900 nm was used for further analysis (Figure 2). Similar to other green plant leaves, the reflectance peak was observed around 550 nm and a reflectance valley was observed between 650 nm and 700 nm. The reflectance value rose between 700 and 750 nm and the reflectance was kept at a high level between 750 nm and 900 nm.

### 3.2. Outlier Detection

**Figure 3:**
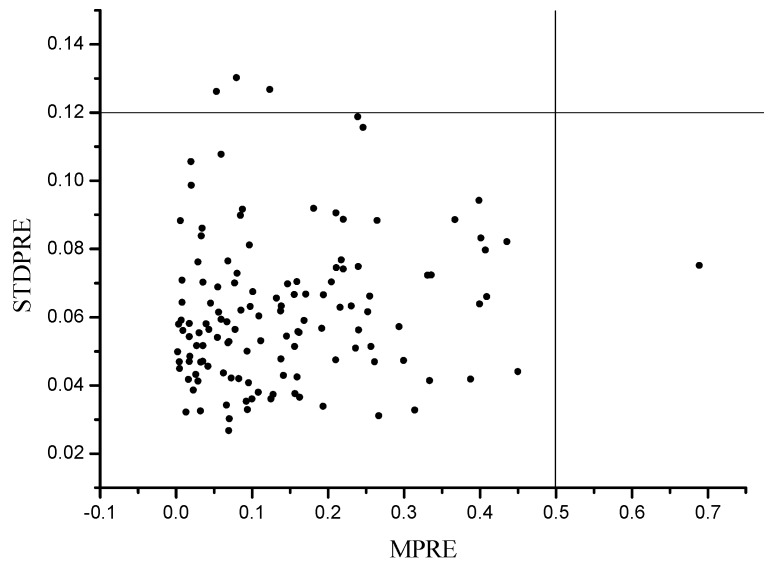
Scatter plot of MPRE and STDPRE of MCPLS.

1000 PLS models were built by randomly selecting 80% samples for calibration and the remaining 20% for prediction. The MPRE and STDPRE of each sample were calculated, and the scatter plot of MPRE-STDPRE was shown in Figure 3. Samples with high MPRE and STDPRE were regarded as outliers. In this study, samples with MPRE over 0.5 and STDPRE over 0.12 were defined as outliers and removed from the sample set. Finally, four samples were removed.

### 3.3. Statistics of Measured Samples

The remaining samples after outlier detection were divided into the calibration set and the prediction set at the ratio of 3:1 of each growth stage. In total, 92 samples were selected as the calibration set and the remaining 32 samples were used as the prediction set. Table 1 showed the statistical results of soluble protein content in oilseed rape leaves.

**Table 1:** Statistics analysis of measured samples of the calibration set and the prediction set.

Sample Sets	Number	Range (mg/g)	Mean (mg/g)	SD (mg/g)
Calibration	92	1.3842–2.9966	2.1811	0.4944
Prediction	32	1.4555–2.9950	2.2262	0.4985

### 3.4. PLS Model Using Full Spectra

**Figure 4:**
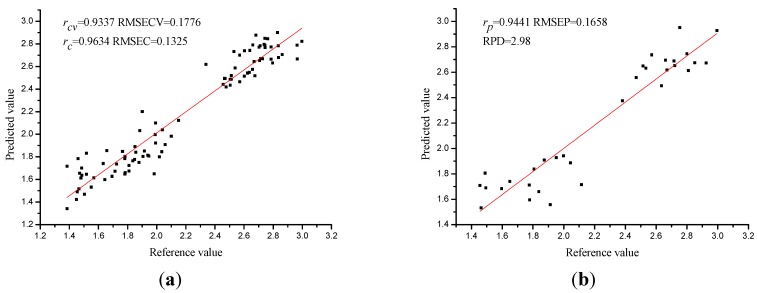
Prediction results of the prediction set (**a**) and the calibration set (**b**) of PLS model using full spectra. (The unit of RMSECV, RMSEC and RMSEP was mg/g in the entire paper).

The prediction of soluble protein content of rape leaves was performed by using PLS with full cross validation. The optimum number of latent variables for the PLS model was 13. The *r_cv_* and RMSECV of the calibration set were 0.9337 and 0.1776 mg/g, respectively, and the cross validation result indicated that hyperspectral imaging could be efficiently used to estimate the soluble protein content of rape leaves. The PLS prediction result was presented in the scatter plot (Figure 4). Moreover, *r_c_* and *r_p_* were both higher than 0.9, and the RPD (residual prediction deviation) value was 2.98 indicated that PLS model was robust in predicting soluble protein content of rape leaves.

### 3.5. Sensitive Wavelengths Selection

To reduce the number of wavelength variables for more reliable, simpler calibration models, weighted regression coefficients (*Bw*), GAPLS, and SPA were applied to select sensitive wavelengths which carried the most information for predicting the quality parameters from the full spectra. The sensitive wavelengths selected by three methods were shown in Table 2. *Bw*, SPA and GAPLS selected 18, 15, 16 sensitive wavelengths, respectively. It was noticed that different sensitive wavelengths were selected by the three methods due to different variable selection principles. While the number of the sensitive wavelengths selected by the three methods were a little different.

**Table 2:** The selected sensitive wavelengths by weighted regression coefficients (*Bw*), SPA and GAPLS.

Methods	Number	Sensitive Wavelengths (nm)
*Bw*	18	501, 508, 542, 707, 720, 739, 761, 769, 789, 809, 852, 859, 865, 871, 880, 892, 897, 899
SPA	15	892, 543, 897, 618, 782, 554, 701, 635, 746, 505, 852, 712, 677, 512, 684
GAPLS	16	788, 789, 809, 636, 638, 778, 807, 639, 635, 738, 791, 810, 866, 679, 741, 777

### 3.6. PLS Models on Selected Wavelengths

**Figure 5:**
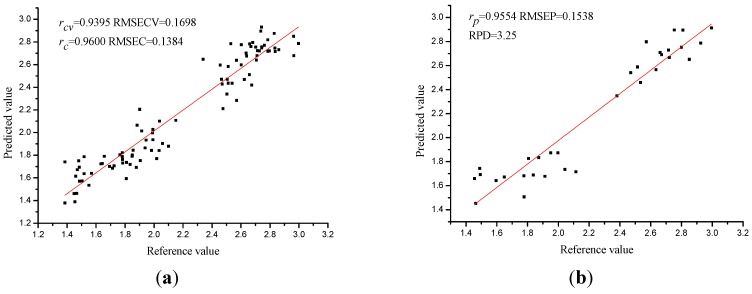
Prediction results of the calibration set (**a**) and the prediction set (**b**) of PLS model using sensitive wavelengths selected by SPA.

As a consequence of the previous analysis, the wavelength selection methods significantly reduced the number of wavelengths. The selected sensitive wavelengths were then used to establish calibration models instead of the full range spectral data. By using the sensitive wavelengths, the performances of PLS models were shown in Table 3 and Figure 5.

**Table 3:** Results of PLS model using sensitive wavelengths selected by weighted regression coefficients (*Bw*), SPA and GAPLS.

Models	LVs	*r_cv_*	RMSECV	*r_c_*	RMSEC	*r_p_*	RMSEP	RPD
*Bw*-PLS	8	0.9095	0.2058	0.9303	0.1813	0.9058	0.2142	2.30
SPA-PLS	12	0.9395	0.1698	0.9600	0.1384	0.9554	0.1538	3.25
GAPLS-PLS	8	0.9288	0.1837	0.9494	0.1553	0.9223	0.1927	2.55

The performances of PLS models varied, but *r_cv_*, *r_c_* and *r_p_* of all PLS models were over 0.9 which indicated the PLS models performed well. *Bw*-PLS, SPA-PLS, GAPLS-PLS obtained the optimal results by constructing the 18, 15 and 16 wavelengths into 8, 12, 8 LVs. *Bw*-PLS obtained relatively worse performance than the other two PLS models, with the RMSECV, RMSEP over 0.2 and RPD of 2.30. Although optimal SPA-PLS obtained more latent variables (12 LVs) with fewer input variables, SPA-PLS performed best with the highest *r_cv_* (0.9395), *r_c_* (0.9600) and *r_p_* (0.9554) and the lowest RMSECV (0.1698 mg/g), RMSEC (0.1384 mg/g), RMSEP (0.1538 mg/g), and the RPD (3.25) of SPA-PLS was over 3 which indicated that SPA-PLS was a robust and accurate calibration model.

The wavelength number was reduced by 96.20%, 96.84%, and 96.62% after wavelength selection by *Bw*, SPA, and GAPLS, which significantly simplified the calibration model and reduced the computation complexity. The performances of the models showed slightly change, *Bw*-PLS and GAPLS-PLS obtained relatively worse performances with less LVs than full spectra PLS while the performance of SPA-PLS was a little better than full spectra PLS with almost the same number of LVs. The results indicated that using variable selection methods to select sensitive wavelengths were efficient for reduction of spectral data as well as the establishment of calibration model.

### 3.7. Visualization of Soluble Protein Content Distribution

**Figure 6:**
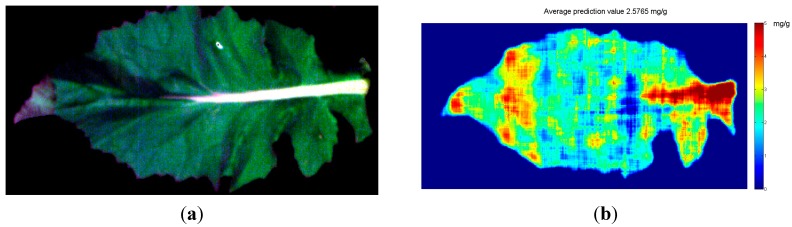
Original RGB image (**a**) and the distribution maps of soluble protein content of oilseed rape leaf (the average predicted value is on the top of the figure) (**b**).

The spectral and spatial information of each pixel in the hyperspectral image made it possible to predict the quality parameters of each pixel by using the calibration models [18,21]. The pixels with similar spectral and spatial information would be predicted to have similar quality parameters, and then these pixels would present similar visualization. Otherwise, the pixels with different characteristics would present different visualizations with different quality parameters. In this study, SPA-PLS obtained the best results with the least sensitive wavelengths, and SPA-PLS was used to estimate the soluble protein content of each pixel. The prediction values of all pixels were calculated by the following equation:

(2) $$Y=BX+B_{0}$$

where *X* was the selected sensitive wavelengths of each pixel, and *B* was the corresponding matrix of the regression coefficient, and *B*_0_ was a constant fitting this model (*B*_0_ = 2.11708 in this study). The visualization map of the predicted soluble protein was achieved and spatially smoothed (shown in Figure 6b), and the average predicted value of soluble protein content in the leaf presented in Figure 6b was 2.5765 mg/g, which was similar to the average reference value of 2.5515 mg/g. The result of the prediction map indicated that using hyperspectral imaging to estimate the soluble protein content of rape leaves was feasible. However, it could be noted that predicted soluble protein contents of some parts exceeded the range of the calibration set and the prediction set, the reason might be: (1) the reference value was the average value of three measurements, and only a small part of the sample was used in each measurement, and the average spectrum of the sample was used to build calibration models; (2) the noises of the hyperspectral image affected the spectrum of each pixel; (3) the leaf vein showed different spectral features from the leaf part, resulting the maximum values in leaf vein in Figure 6b. For future study of visualization of soluble protein content distribution, the shortcomings should be overcome by improving the model performances with wider reference value range and minimum noises of the spectrum.

## 4. Conclusions

A hyperspectral imaging system covering the spectral range 380–1030 nm was built to estimate the soluble protein content of rape leaves. PLS was used to build calibration models using the 500–900 nm spectra and the soluble protein content of the rape samples. PLS models obtained good performance by using full spectra and the optimal wavelengths selected by *Bw*, SPA, and GAPLS. SPA-PLS obtained the best performance and SPA-PLS was used to form the distribution map by predicting the soluble protein content of each pixel. The results obtained in this study indicated that hyperspectral imaging combined with wavelength selection methods and multivariate calibration methods could estimate the soluble protein content of rape leaves and visualize the prediction map of soluble protein of rape leaves. For further study, more rape leaves with a wider range of soluble protein content should be studied to build more robust and global calibration models and select more reliable sensitive wavelengths. More efforts were needed to set up multispectral imaging systems for on-line estimation and visualization of soluble protein content of rape leaves. The hyperspectral imaging system applied in this study was laboratory based system, and it was mainly used for leaf scale measurement. The hyperspectral imaging system for on-line field application should be developed to study soluble protein measurement and distribution for both canopy scale and leaf scale in the future.
